# Patient reported outcome measures (PROMs) in primary care: an observational pilot study of seven generic instruments

**DOI:** 10.1186/1471-2296-15-88

**Published:** 2014-05-06

**Authors:** Jan-Willem Weenink, Jozé Braspenning, Michel Wensing

**Affiliations:** 1 Scientific Institute for Quality of Healthcare, Radboud University Medical Center, P.O. Box 9101, 6500 HB Nijmegen, the Netherlands

**Keywords:** Patient-reported outcome measures, Primary care, General practice, Quality of life, Patient empowerment, Patient enablement

## Abstract

**Background:**

Patient reported outcome measures (PROMs) have been introduced in studies to assess healthcare performance. The development of PROMs for primary care poses specific challenges, including a preference for generic measures that can be used across diseases, including early phases or mild conditions. This pilot study aimed to explore the potential usefulness of seven generic measures for assessing health outcomes in primary care patients.

**Methods:**

A total of 300 patients in three general practices were invited to participate in the study, shortly after their visit to the general practitioner. Patients received a written questionnaire, containing seven validated instruments, focused on patient empowerment (PAM-13 or EC-17), quality of life (EQ-5D or SF-12), mental health (GHQ-12), enablement (PEI) and perceived treatment effect (GPE). Furthermore, questions on non-specific symptoms and number of GP contacts were included. After 4 weeks patients received a second, identical, questionnaire. Response and missing items, total scores and dispersion, responsiveness, and associations between instruments and other measures were examined.

**Results:**

A total of 124 patients completed the questionnaire at baseline, of whom 98 completed it both at baseline and 4 weeks later (response rate: 32.7%). The instruments had a full completion rate of 80% or higher. Differences between baseline and follow up were significant for the EQ-5D (p = 0.026), SF-12 PCS (p = 0.026) and the GPE (p = 0.006). A strong correlation (r ≥ 0.6) was found between the SF-12 MCS and GHQ-12, at both baseline measurement and after four weeks. Other observed associations between instruments were moderately strong. No strong correlations were found between instruments and non-specific symptoms or number of GP contacts.

**Conclusions:**

The present study is among the first to explore the use of generic patient-reported outcome measures in primary care. It provides several leads for developing a generic PROM questionnaire in primary care as well as for potential limitations of such instruments.

## Background

Patient reported outcome measures (PROMs) are standardised, validated questionnaires that are completed by patients to measure perceived health status, functional status or health-related quality of life [1]. While PROMs are used in health research to document health outcomes, in particular treatment effectiveness in clinical trials [2], today they are also used to measure healthcare quality. For instance, in 2009 the National Health System in the UK started to use PROMs to assess the quality of four elective procedures [3]. The adoption of PROMs in primary care, however, poses specific challenges that are related to the specific characteristics of their patient population. Primary care patients show a wide range of diseases, including many early undifferentiated stages and mild conditions. Furthermore, primary care provides comprehensive and continuing healthcare [4]. From the WONCA competencies and corresponding characteristics of general practice follow some other domains that can be measured at the patient level and may be appropriate as outcome measures. These are that the general practitioner should “develop a person centred approach orientated to the individual, his/her family, their community, where it as important to understand how the patient copes with and views their illness as dealing with the disease process itself”; and that the general practitioner should “promote patient empowerment” [4]. Scales measuring patient enablement and patient empowerment may be appropriate to measure these domains. When developing PROMs for primary care these factors should be taken into account, implying that generic measures that can be used across diseases are preferable to disease-specific measures, and that a broad set of domains of general practice should be addressed by a PROM.

Many questionnaires exist that aim to assess primary care performance from the patients’ perspective. For instance, the Primary Care Assessment Survey (PCAS) studies seven domains of general practice, such as accessibility, continuity, comprehensiveness and interpersonal treatment [5]. The European Task Force on Patient Evaluations of General Practice (EUROPEP) measures patient evaluations of a broad range of specific aspects of general practice care, such as showing interest, involving the patient in decision making and thoroughness [6], and the Patient Assessment of Chronic Illness Care (PACIC) studies chronic care delivery [7]. Most existing questionnaires for assessing primary care performance, however, focus on the organisation and process of healthcare delivery, instead of care outcomes.

Some validated questionnaires for functional status or quality of life, which were not primarily developed for primary care performance measurement, may be good options for PROMs in primary care. Before embarking on the development of a new tool, we explored a number of existing measures that focus on these domains in a pilot study. Besides its generic character, we felt that a potentially useful PROM should have high relevance for primary care patients (indicated by good response rates), have potential to discriminate between care providers (indicated by absence of highly skewed distributions), show responsiveness to change of patients’ symptoms over time, and be predictably correlated with other measures. Based on these predefined criteria, we aimed to explore the potential usefulness of seven generic patient reported outcome measures in primary care. The results of this pilot study can possibly be used to inform further research and development of PROMs in primary care as well as for reflection on the potential limitations of PROMs in primary care.

## Methods

### Design and setting

An observational study was performed in patients who visited their general practitioner for consultation in one of three participating practices (five general practitioners in total). A maximum of 60 patients per general practitioner was invited to minimize workload for general practitioners. Practices were situated in the south-eastern part of The Netherlands, and concerned one practice in an urban area and two in a rural area. One practice was single-handed and two were group practices. Ethical approval was received for this study from the Arnhem-Nijmegen ethical committee.

### Study population

A total of 300 patients was invited who visited one of the participating general practitioners for a consultation. Patients were not invited to participate if they were younger than 18 years old, terminally ill, or had psychological problems or a mental handicap as a result of which the GP estimated the patient was not suitable to participate in research at the moment. Written questionnaires were handed out by the general practitioner during the consultation. Patients were asked to complete the questionnaire and return it to the research institute in a pre-paid envelope. In the questionnaire, patients were asked if they were willing to complete a second identical questionnaire after 4 weeks. If so, patients were sent a second questionnaire by the research institute.

### Measures

We performed a comprehensive search in PubMed using keywords primary care and patient reported outcomes. We scanned articles and references of relevant articles for existing questionnaires on the outcome domains listed in Table 1. Furthermore, we consulted colleagues to identify instruments they had previously used. We searched the internet for a Dutch translation of questionnaires, and only included questionnaires that were available in Dutch. For some domains multiple questionnaires were found. We excluded questionnaires on the basis of length. The selected questionnaires are listed in Table 2, and are further elucidated on in the paragraphs below. Excluded questionnaires included the Measure Yourself Medical Outcome Profile (MYMOP) [8] and the Outcome Related Impact on Daily Life (ORIDL) [9] for unavailability of a Dutch translation, and the Sickness Impact Profile (SIP) [10] due to its length. Furthermore, the Spielberger State-Trait Anxiety Inventory (STAI) [11] was not included since it focuses specifically on anxiety. We chose to include generic instruments focusing on mental health, thereby limiting the total length of our questionnaire. Finally, we included a Global Perceived Effect scale (GPE) for assessing the effect of received care.

**Table 1:** Possible outcome domains for PROMS following from the WONCA definition of general practice

**WONCA competency**	**WONCA characteristics**	**Possible outcome domain**
Comprehensive approach	Promotes health and well-being	General-, physical and mental health
Person-centred care	Promotes patient empowerment	Patient empowerment
	Centred on patient and context	Patient enablement

**Table 2:** Domains and used questionnaires

**Domain**	**Questionnaire**
Patient Empowerment	Patient Activation Measure (PAM-13)
	Effective Consumer Scale (EC-17)
General Health	EuroQol 5D (EQ-5D, including EQ-VAS)
	Short Form 12 (SF-12)
Mental Health	General Health Questionnaire (GHQ-12)
Enablement	Patient Enablement Instrument (PEI)
Effect	Global Perceived Effect (GPE)

The Patient Activation Measure (PAM-13) and the Effective Consumer Scale (EC-17) were alternately used to measure patient empowerment. The PAM-13 consists of 13 items that evaluates a patient’s knowledge, skills and confidence to manage their own health [12]. Item scores are converted in one activation score, reflecting a patient’s activation level. Missing values are accounted for in calculation of the total score. The EC-17 consists of 17 items on 5 subscales (use of health information, clarifying priorities, communication with others, negotiating own role, and taking action) [13]. Item scores are converted to a score on a 0-100 scale. If more than 3 items are missing no total score is computed. Because the EC-17 specified *having a disease* in its questions, we added a *not applicable* response option, which we treated as missing data in computing the total score.

The EuroQol-5D (EQ-5D) and the Short Form 12 (SF-12) were alternately used to measure quality of life. The EQ-5D consists of five dimensions (mobility, self-care, usual activities, pain/discomfort and anxiety/depression) with three response categories (no problems, some problems, extreme problems) [14]. Total score was calculated using Dutch population norms [15]. Furthermore, the EQ-5D contains a visual analogue scale (VAS) on which respondents score their current health status on a scale from 0 to 100. The SF-12 is a 12-item questionnaire measuring eight domains: physical functioning, role-physical, bodily pain, general health, vitality, social functioning, role-emotional and mental health [16]. Each item is scored on a 3- or 5-point Likert scale, and for each domain a total score is computed on a 0-100 scale. With these item scores, a physical component summary (PCS) and a mental component summary (MCS) can be calculated.

The General Health Questionnaire (GHQ-12) was used to measure mental health. It consists of 12 items, each with a 4-point response category (from ‘better than usual’ to ‘much less than usual’) [17], in which each item receives a score of 0, 1, 2 or 3. The total score for the GHQ-12 thus ranges from 0 to 36, where a lower score reflects better mental health.

The Patient Enablement Instrument (PEI) was used to measure patient enablement [18]. It consists of six items, each with three response categories (0 = ‘same or worse’, 1 = ‘better’, 2 = ‘much better’). The range of the aggregated sum score is 0 to 12, with a higher score indicating a higher level of enablement.

A Global Perceived Effect (GPE) scale was used to measure perceived effect of treatment. The scale consists of one item that asks patients about perceived effect of treatment [19]. Patients score on a seven point response scale (with 1 = ‘worse than ever’, 7 = ‘fully recovered’). Furthermore, we dichotomized the GPE scores into “improved” (“completely recovered” and “much improved”) versus “not improved” (“slightly improved”, “not changed”, “slightly worsened”, “much worsened”, “worse than ever”), and added a question about treatment satisfaction (also on a seven-point scale).

The questionnaire also included questions about non-specific symptoms and the number of GP contacts in the previous 12 months. Non-specific symptoms included fatigue, dizziness, headache, weakness, palpitation and sleep problems, and their presence can indicate underlying changes in emotional well-being [20]. As said, some instruments that aimed to measure the same domain were used alternately to reduce length. Therefore, a total of four versions of the questionnaire were used.

### Sample size calculation

For our explorative study, no figures were available for a proper power calculation given the new context of the questionnaires distributed. We based our sample size largely on what number of questionnaires would be feasible in terms of burden for the recruiting GPs and what sample size would give a meaningful precision. Therefore, we invited a total of 300 patients (60 per GP). We expected a response rate of approximately 50%, resulting in all instruments having a response of 150 patients or 75 patients (in case of the EQ-5D, SF-12, PAM-13 and EC-17). This results in a precision (half width of the 95%-CI for mean difference) of 4.8 and 3.5 points (assuming a 100 points scale with a SD of at most 15), which seemed sufficiently precise to detect non-small differences [21].

### Data-analysis

We first studied the response on individual instruments, and the missing values on items. Instruments with a low response or a high number of missing scores were considered less appropriate for potential use in practice.

Secondly, we studied statistical dispersion of scores for each of the instruments, exploring mean, minimum and maximum scores and standard deviations. We examined if data was normally distributed by exploring histograms and using the Shapiro-Wilk test. Furthermore, we studied floor and ceiling effects in terms of percentage of patients using the most extreme (upper or lower) response categories. Instruments with a squeezed distribution, or high presence of floor and ceiling effects were thought to be less appropriate for potential use in practice.

Responsiveness has been defined as the ability of an instrument to accurately detect change when it has occurred [22]. Change in instrument scores between baseline and follow-up were explored, and were tested on significance with a paired samples t-test or in case of a skewed distribution with a Wilcoxon signed-rank test. We explored changes in scores between baseline and follow up, where positive changes could reflect the relief of complaints at baseline due to treatment or favourable natural development. Furthermore, we used Pearson correlation to identify moderate (r = .40-.59), strong (r = .60-.79) and very strong associations (r = .80 = 1.0) between instrument scores [23]. We explored if scores of instruments focusing on the same domains correlated.

Finally, we looked at treatment satisfaction, presence of non-specific symptoms and number of contacts with the GP to assess construct validity. Construct validity refers to the extent to which scores on a particular instrument relate to other measures in a manner that is consistent with theoretically derived hypotheses concerning the concepts that are being measured [24,25]. We expected that treatment satisfaction would be positively correlated with instruments scores [26], while the presence of non-specific symptoms was expected to have a negative correlation with instrument scores [20]. Furthermore, we explored if visiting frequency was associated with instruments scores. Since all seven instruments had previously been validated in other contexts, we expected that content validity was assured.

Finally, an a posteriori sample size calculation was performed to learn about the number of questionnaires needed to show a meaningful difference.

## Results

Of 300 invited patients, 124 completed the questionnaire at baseline and 98 patients completed the questionnaire both at baseline and after 4 weeks (response rate: 32.7%). Response percentages ranged from 16.7% to 50.0% across the participating general practitioners. Table 3 provides descriptive information of the study population. In comparison with Dutch GP population, our study population was less ethnically diverse and more likely to have one or more chronic illnesses [27]. Most prevalent chronic illnesses were cardiovascular disease (31.6%), diabetes (15.3%) and depressive symptoms (11.2%). 56% of patients with a chronic illness used medication.

**Table 3:** Patients’ characteristics (n = 98)

	**Study population**
Mean age (SD)	62.5 (12.2)
Percentage women	44.2%
Percentage with higher education	34.7%
Percentage with single household	18.3%
Percentage of Dutch decent	99.0%
Percentage with one or more chronic illnesses	55.1%^a^

^a^Included asthma, cardiovascular disease, COPD, depression and diabetes.

### Response and missing items

Response percentages and the number of missing items on the individual instruments are presented in Table 4. The response on the different instruments ranged from 87.5% to 99.0%. Of each instrument, over 80% was completed without any missing items. The EC-17 had a relatively high number of missing values. The response on the EC-17 was 91.8%, but because we treated the *not applicable* response option as missing and no total score was computed with more than 3 missing items, a total of 36 scores remained (73.5%).

**Table 4:** Response on individual measures

	**Number of items**	**Completed at baseline and after 4 weeks (n)**	**Baseline measurement**				**After 4 weeks**			
			**0 missing**	**1 missing**	**2 missing**	**≥3 missing**	**0 missing**	**1 missing**	**2 missing**	**≥3 missing**
PAM-13	13	95.9% (47/49)	43	4	-	-	45	2	-	-
EC-17^a^	17	91.8% (45/49)	43	1	1	-	39	5	1	-
EQ-5D	5	94.0% (45/50)	42	3	-	-	43	2	-	-
EQ- VAS^b^	1	94.0% (47/50)	47				47			
SF-12	12	87.5% (42/48)	42	-	-	-	42	-	-	-
GHQ-12	12	99.0% (97/98)	94	2	1	-	94	3	-	-
PEI	6	99.0% (97/98)	94	1	-	2	92	-	2	3
GPE^b^	1	90.8% (89/98)	89				89			

^a^If 3 or more items were missing or not applicable no total score was computed; 36 total scores computed (73.5%).

^b^Instrument consists of 1 item.

### Dispersion

The median, minimum and maximum scores, as well as inter-quartile ranges (IQR) at baseline and after four weeks are presented in Table 5. Floor and ceiling effects for the specific measures are provided in Table 6. In comparison to other instruments, the EQ-5D had a high prevalence of maximum scores and the PEI had a high prevalence of minimum scores.

**Table 5:** Total scores and change of scores on individual measures (n = 98)

		**Baseline score**				**Score after four weeks**				**Score difference**			
**Instrument (n)**	**Theoretical range of scale**	**Median**	**Min.**	**Max.**	**IQR**	**Median**	**Min.**	**Max.**	**IQR**	**Median**	**Min.**	**Max.**	**IQR**
PAM-13 (47)	0 - 100	56.4	41.7	100.0	49.9 - 70.8	56.4	40.1	100.0	49.9 - 70.8	0.0	−43.6	38.9	−2.8 to 6.7
EC-17 (36)^a^	0 - 100	76.5	55.9	100.0	75.0 - 83.8	80.3	63.2	98.5	75.0 - 88.1	0.0	−10.9	36.8	−2.4 to 4.4
EQ-5D (45)	0 - 1	0.84	0.15	1.00	0.79 - 0.89	0.84	0.25	1.00	0.81 - 1.0	0.00^c^	−0.44	0.28	0.00 to 0.11
EQ- VAS (47)	0 - 100	70.0	30	100	60.0 - 80.0	75.0	40	100	70.0 - 80.0	0.0	−25.0	20.0	−5.0 to 10.0
SF-12 PCS (42)	0 - 100	42.3	18.7	60.5	32.4 - 53.5	49.8	17.8	58.9	37.5 - 54.4	2.0^c^	−15.3	28.8	−1.3 to 7.1
SF-12 MCS (42)	0 - 100	56.0	17.3	65.8	48.7 - 59.0	54.1	19.8	62.7	49.3 - 58.9	0.0	−25.8	20.9	−6.3 to 4.4
GHQ-12 (97)	0 - 36^b^	9.0	3.0	26.0	7.0 - 13.0	8.0	3.0	25.0	7.0 - 12.0	0.0	−13.0	10.0	−2.0 to 2.0
PEI (97)	0 - 12	0.0	0.0	6.0	0.0 - 2.5	0.0	0.0	12.0	0.0 - 2.0	0.0	−6.0	8.0	−0.5 to 0.0
GPE (89)	1 - 7	4.0	2	7	4.0 - 5.0	4.0	3	7	4.0 - 6.0	0.0 ^c^	−3.0	3.0	0.0 to 1.0

^a^If 3 or more items missing then no score was computed.

^b^Lower score means better health.

^c^Mean score difference significant with p ≤ 0.05.

**Table 6:** Floor and ceiling effects

**Instrument (n)**	**Minimum score baseline**	**Maximum score baseline**	**Minimum score follow up**	**Maximum score follow up**
PAM-13 (47)	0.0% (0/47)	2.1% (1/47)	0.0% (0/47)	2.1% (1/47)
EC-17 (36)	0.0% (0/36)	2.8% (1/36)	0.0% (0/36)	0.0% (0/36)
EQ-5D (45)	0.0% (0/45)	22.2% (10/45)	0.0% (0/45)	44.4% (20/45)
EQ- VAS (47)	0.0% (0/47)	2.1% (1/47)	0.0% (0/47)	6.4% (3/47)
SF-12 PCS (42)	0.0% (0/42)	0.0% (0/42)	0.0% (0/42)	0.0% (0/42)
SF-12 MCS (42)	0.0% (0/42)	0.0% (0/42)	0.0% (0/42)	0.0% (0/42)
GHQ-12 (97)	0.0% (0/97)	0.0% (0/97)	0.0% (0/97)	0.0% (0/97)
PEI (97)	54.6% (53/97)	0.0% (0/97)	60.8% (59/97)	1.0% (1/97)
GPE (89)	1.1% (1/89)	5.6% (5/89)	0.0% (0/89)	6.7% (6/89)

### Responsiveness

All measures showed increased mean scores over time, indicating improvement in health status, though for most instruments no median score differences were observed. The differences in mean scores between baseline and follow up at four weeks were significant for the EQ-5D (p = 0.026), SF-12 PCS (p = 0.026) and the GPE (p = 0.006). When looking at dichotomous scores of the GPE, we found that 15 out of 89 patients at baseline right after the consultation, and 27 out of 89 patients after four weeks indicated to have improved after their visit to the GP. A total of 18 patients improved after four weeks in relation to baseline, while 6 patients worsened in these four weeks. Table 7 presents the percentage of patients that had an increased or worsened score for the specific measures. In comparison to other measures, the EQ-5D and PEI showed little change across time with approximately half of the patients having the same score at follow-up.

**Table 7:** Improved scores on individual measures (% of patients)

**Instrument (n)**	**Improved score**	**Same score**	**Worsened score**
PAM-13 (47)	46.8%	14.9%	38.3%
EC-17 (36)	47.2%	19.4%	33.3%
EQ-5D (45)	40.0%	46.7%	13.3%
EQ- VAS (47)	48.9%	21.3%	29.8%
SF-12 PCS (42)	57.1%	7.1%	35.7%
SF-12 MCS (42)	47.6%	7.1%	45.2%
GHQ-12 (97)^a^	47.4%	11.3%	41.2%
PEI (97)	22.7%	52.6%	24.7%
GPE (89)	41.6%	37.1%	21.3%

^a^Lower total score on the GHQ reflects improved score.

### Associations between instruments

For baseline scores, strong associations were found between the SF-12 MCS and the GHQ (r = -0.768, p = 0.000), and the EQ-5D en EQ-VAS (r = 0.604, p = 0.000). Moderate associations were found between the PAM-13 and EQ-5D (r = 0.409, p = 0.043), the PAM-13 and SF-12 MCS (r = 0.438, p = 0.079), and the EQ-VAS and GPE (r = 0.429, p = 0.004). For scores after four weeks, strong associations were found between the EC-17 and EQ-VAS (r = 0.709, p = 0.010), EQ-VAS and GPE (r = 0.661, p = 0.000), the GHQ and SF-12 MCS (r = -0.705, p = 0.000), and the GHQ and EQ-VAS (r = -0.633, p = 0.000). Moderate associations were found between the PAM-13 and EQ-5D (r = 0.440, p = 0.028), PAM-13 and EQ-VAS (r = 0.552, p = 0.003), PAM-13 and GPE (r = 0.481, p = 0.001), EQ-5D and EQ-VAS (r = 0.568, p = 0.000), EQ-5D and GPE (r = 0.542, p = 0.000), EC-17 and GHQ (r = -0.492, p = 0.002), GHQ and SF-12 PCS (r = -0.420, p = 0.006). When looking at change of scores on the instruments, strong associations were found between the EC-17 and EQ-VAS (r = 0.554, p = 0.061), SF-12 PCS and SF-12 MCS (r = -0.523, p = 0.000), and GHQ and SF-12 MCS (r = -0.530, p = 0.000). A moderate association was found between the EQ-5D and EQ-VAS (r = 0.438, p = 0.003).

### Associations with other measures

At baseline, a total of 77 out of 91 patients reported to be very or absolutely satisfied with treatment (84.6%). At follow-up this was 69 patients (75.9%). A moderate positive correlation was found between treatment satisfaction and the EC-17 (r = 0.490, p = 0.003) at baseline. At follow-up, a strong correlation was found between treatment satisfaction and the SF-12 PCS (r = 0.575, p = 0.000). No other significant correlations were found.

At baseline, 69.4% of the patients reported to have suffered from one or more non-specific symptoms in the past four weeks. These included fatigue (57.3%), headache (36.0%) and sleep problems (34.8%). After four weeks this was 70.4%, with fatigue (53.9%), headache (37.5%) and sleep problems (26.4%) most often mentioned. A total of 59 patients (60.2%) indicated at both measure moments to have suffered from one or more non-specific symptoms in the past four weeks. A moderate negative association was found between the presence of non-specific systems and the SF-12 PCS score (r = -0.424, p = 0.005) after four weeks, though at baseline no significant association was found (r = -0.272, p = 0.082).

The mean number of reported GP contacts in the past 12 months was 6.9 at baseline. At follow-up, patients reported to have had an average of 1.3 GP contacts in the four weeks between baseline and follow-up. No correlations were found between the number of GP contacts and instrument scores.

### Number of questionnaires needed

Most instruments in our study had a SD between 10-15% of the instruments’ range, resulting in a required sample size of N = 400 to detect small differences between baseline and after four weeks [21].

## Discussion

We found high completion rates for all seven instruments, with only a small number of items missing. Total scores for the instruments varied across patients, with the EQ-5D and PEI having a relatively high prevalence of maximum and minimum scores respectively, and most instruments being susceptible for change in the period between baseline and after four weeks. Some strong associations were found between the seven instruments, and between instruments and other measures such as treatment satisfaction and non-specific symptoms, but overall correlations tended to be weak or moderate. Based on our predefined criteria none of the seven instruments seem to stand out in a positive or negative way, and their potential use as PROMs should be studied more elaborately. Finally, the low response rate needs to be considered if PROMs are used in performance measurement systems, because this could lead to selection bias.

Our study is one of the first to explore the use of generic patient-reported outcome measures in primary care. In the US, the Patient-Reported Outcome Measurement Information Systems (PROMIS) aims at the continuing development of patient-reported measures that are comparable across studies and diseases [28]. These measures focus on the domains physical-, mental- and social health, and in the current literature on PROMs we also see a focus on quality of life. The present study adds that it explores a broad set of outcome domains (i.e. empowerment, mental health, physical health, general health, enablement and perceived treatment outcome) that all seem to be of importance in primary care.

The present study had a low response compared to recent studies conducted in Dutch general practice [29,30]. This low response may indicate selection bias, making it uncertain whether the sample reflected the general practice population. If such a measure were to be used as a performance measure, a low response would have its implications on interpreting the data. In our study we did not send a reminder, because we obtained patients’ contact information only after their return of the baseline questionnaire. One potential explanation for the low response rate is the length of the questionnaire. Shortening the measure might result in an increased response in future studies, as has been demonstrated in previous studies [31]. The relatively small size of the study limited the possibility to detect small differences in time or between groups of patients, and significant associations between instruments and other measures. This makes it hard to draw firm conclusions from this study regarding the seven instruments, and replication in larger studies is required with a sample size of at least 400 patients. Despite these limitations, the study provided a number of important leads to the further development of PROMs for adoption in primary care.

Ideally, PROMs are measured before and after a specific intervention. In general practice, however, it is often difficult to determine a clear start and endpoint of treatment. In this study we had two measure moments, both after the consultation with the physician. Therefore the change may reflect effectiveness of interventions, natural course of symptoms, or measurement error. Because continuity of care is one of the hallmarks of general practice, interventions are not limited to one episode of care but cover patient’ health needs longitudinally [4,32]. The data therefore could still express performance of general practice. Further research is needed to determine if other measure moments than those used in the present study are favourable in primary care.

The seven included instruments were frequently subject of study in previous research, though only limited as outcome measures in the setting of a generic population in general practice.

In our study we found a low responsiveness to change of the EQ-5D, also reflected by a high prevalence of maximum scores at both baseline and after four weeks. Previous studies showed ambiguous results regarding responsiveness of the EQ-5D [33,34]. This might be explained by the different settings in which these studies took place. Our findings suggest that for a generic population visiting the GP other instruments that measure quality of life such as the SF-12 might be more appropriate, though no firm conclusions can be drawn.

The EC-17 specifically focuses on measuring main skills and behaviours needed to effectively manage ones chronic disease. Some of the items of the EC-17 are explicitly targeted at the patients’ disease. This resulted in a relatively low number of applicable answers on this instrument, since not all patients in our study population had a disease. The PAM-13 also focuses on chronic patients, though items are targeted at the patients’ health instead of the patients’ disease, which might explain why this instrument resulted in a higher response rate. This might opt for including the PAM-13 for measuring empowerment, though its validation for a general population in the primary care setting needs be studied.

Previous research that studied the outcome of patient consultations found associations between some of the used instruments and other measures, such as the PEI and the patients’ health status [35] and the PEI and treatment satisfaction [18], showed ambiguous results regarding the relation between health status and treatment satisfaction [26,36], and related the presence of non-specific symptoms to emotional distress [20]. In our study we only found a few strong associations, such as that between the GHQ and SF-12 MCS scores, which was to be expected since they both measure mental health, and between treatment satisfaction and the physical component scale of the SF-12. No other strong associations were found between instruments, or with other measures.

This study is to our knowledge one of the first that studies several previously validated questionnaires on different domains as potential PROMs in primary care. It may be used in the further exploration of adapting PROMs in general practice, though our findings are only preliminary results and further research is needed. We think that embedding a short informative measure in the care delivery process where it acts as a feedback tool on the patients’ level brings along opportunities*.* This way the added value for both GP and patient is clear, and it is easier for the GP to act upon this feedback in daily practice if needed. On the other side, embedding PROMs in the care process increases workload for the GP, which needs to be taken into consideration. The potential use of the used instruments as an individual feedback tool in the primary care setting should be studied more elaborately as well. Further research is needed to determine the psychometric properties of previously validated instruments in the current setting of study (i.e. generic primary health care population). Finally, the relation between the studied instruments with relevant clinical measures and the quality of delivered care is a point of interest for future studies.

## Conclusions

This study showed that several generic instruments on the domains of quality of life, patient empowerment and patient enablement might be fit for use as a PROM in primary care, though further research is needed to study their validity in primary care.

## Competing interests

The authors declare that they have no competing interests.

## Authors’ contributions

JW designed the study, was responsible for data collection and data analysis, and wrote the paper. JB and MW designed the study, supervised data-collection and data-analysis, and contributed to the paper. All authors have read and approved the final manuscript.

## Pre-publication history

The pre-publication history for this paper can be accessed here:

http://www.biomedcentral.com/1471-2296/15/88/prepub
